# FZD2 regulates limb development by mediating β-catenin-dependent and -independent Wnt signaling pathways

**DOI:** 10.1242/dmm.049876

**Published:** 2023-03-24

**Authors:** Xuming Zhu, Mingang Xu, N. Adrian Leu, Edward E. Morrisey, Sarah E. Millar

**Affiliations:** ^1^ Black Family Stem Cell Institute, Icahn School of Medicine at Mount Sinai, New York, NY 10029, USA; ^2^ Department of Cell, Developmental and Regenerative Biology, Icahn School of Medicine at Mount Sinai, New York, NY 10029, USA; ^3^ Department of Biomedical Sciences, School of Veterinary Medicine, University of Pennsylvania, Philadelphia, PA 19104, USA; ^4^ Department of Medicine, University of Pennsylvania, Philadelphia, PA 19104, USA; ^5^ Department of Dermatology, Icahn School of Medicine at Mount Sinai, New York, NY 10029, USA; ^6^ Tisch Cancer Institute, Icahn School of Medicine at Mount Sinai, New York, NY 10029, USA

**Keywords:** *Fzd2*, Wnt, Limb, Robinow syndrome, Omodysplasia

## Abstract

Human Robinow syndrome (RS) and dominant omodysplasia type 2 (OMOD2), characterized by skeletal limb and craniofacial defects, are associated with heterozygous mutations in the Wnt receptor FZD2. However, as FZD2 can activate both canonical and non-canonical Wnt pathways, its precise functions and mechanisms of action in limb development are unclear. To address these questions, we generated mice harboring a single-nucleotide insertion in *Fzd2* (*Fzd2^em1Smill^*), causing a frameshift mutation in the final Dishevelled-interacting domain. *Fzd2^em1Smill^* mutant mice had shortened limbs, resembling those of RS and OMOD2 patients, indicating that *FZD2* mutations are causative. *Fzd2^em1Smill^* mutant embryos displayed decreased canonical Wnt signaling in developing limb mesenchyme and disruption of digit chondrocyte elongation and orientation, which is controlled by the β-catenin-independent WNT5A/planar cell polarity (PCP) pathway. In line with these observations, we found that disruption of FZD function in limb mesenchyme caused formation of shortened bone elements and defects in Wnt/β-catenin and WNT5A/PCP signaling. These findings indicate that FZD2 controls limb development by mediating both canonical and non-canonical Wnt pathways and reveal causality of pathogenic *FZD2* mutations in RS and OMOD2 patients.

## Teaser

**Summary:** Limb defects in Robinow syndrome are associated with mutations in FZD2. Using mouse models, we show that FZD2 is causative, and controls limb development via both canonical and non-canonical Wnt pathways.

## INTRODUCTION

Congenital limb defects (CLDs) are common in human populations (Ephraim et al., 2003). Genetic defects are a major cause of CLDs and have been characterized by next-generation sequencing (Carli et al., 2013; White et al., 2018). Although the genetic defects causing CLDs are quite diverse, many of these affect specific signaling pathways. For example, mutations in Wnt/planar cell polarity (PCP) pathway components have been identified in patients suffering from Robinow syndrome (RS), which is characterized by shortened limbs as well as craniofacial defects (White et al., 2018).

Wnt signaling pathways play key roles in many developmental processes and human diseases (Klaus and Birchmeier, 2008). Activation of the canonical pathway is initiated by the binding of Wnt ligands such as WNT1 and WNT3 to a specific subset of Frizzled (FZD) receptors, causing stabilization of cytoplasmic β-catenin and its translocation to the nucleus, where it activates transcription of downstream target genes such as *Axin2* (MacDonald et al., 2009). Non-canonical Wnt pathways are triggered by WNT5A and WNT11, which regulate cytoskeletal arrangement, cell orientation and cell movements, via the PCP or Ca^2+^ pathways (Rim et al., 2022).

In vertebrates, at least 15 Wnt family members are expressed in developing limbs (Witte et al., 2009). Multiple lines of evidence demonstrate that Wnt/β-catenin signaling is essential for limb development. Deletion of *Wnt3* or *Ctnnb1* in mouse limb ectoderm causes failure of normal formation of the apical ectodermal ridge (AER) and limb agenesis (Barrow et al., 2003). Consistent with this, mutations of human *WNT3* are associated with tetra-amelia syndrome, characterized by severe defects in limb development (Niemann et al., 2004). Specific deletion of β-catenin in limb mesenchyme disrupts AER integrity and induces apoptosis of mesenchymal cells (Hill et al., 2006). Mouse genetic studies have also revealed critical roles for WNT/PCP signaling in limb development; for instance, deletion of the *Wnt5a*, *Ror2* or *Vangl2* genes prevents proper elongation and orientation of differentiating chondrocytes along the proximal–distal (P-D) axis of the limb (Gao et al., 2011; Yamaguchi et al., 1999).

Although the roles of WNT ligands and their downstream pathways in limb development have been intensively investigated, the functions of specific FZD transmembrane receptors in this process are less clear. FZD proteins directly bind Wnt ligands, and their intracellular C-terminal domains interact with Dishevelled (DVL) proteins to transduce canonical or non-canonical Wnt signaling (Gordon and Nusse, 2006). Accumulating evidence suggests that activation of canonical versus non-canonical signaling by FZD receptors depends on their binding to specific Wnt ligands. For example, FZD2 interacts with WNT3 and WNT3A to stabilize β-catenin and activate the canonical Wnt pathway in a myeloid progenitor cell line (Dijksterhuis et al., 2015). By contrast, FZD2 interacts with WNT5A to mediate non-canonical Wnt signaling and stimulate epithelial–mesenchymal transition and migration in a variety of tumor cell lines (Gujral et al., 2014).

Human genetic studies provide important contributions for identifying candidate disease genes, and for revealing potential mechanistic relationships in cases in which mutations in different genes are associated with similar disease phenotypes. However, these studies are necessarily correlative. RS and omodysplasia (OMOD) are human syndromes that share a constellation of phenotypes predominantly characterized by shortened limbs and craniofacial defects, as well as variable defects in genitalia, and ear shape and position, among other abnormalities (White et al., 2018; Zhang et al., 2022; Arabzadeh et al., 2022). Autosomal-dominant RS (AD-RS) is associated with heterozygous mutations in *WNT5A* and in the DVL family genes *DVL1*, *DVL2* and *DVL3*, while recessive RS (R-RS) is associated with homozygous mutations in receptor tyrosine kinase-related 2 (*ROR2*) and nucleoredoxin (*NXN*) (Person et al., 2010; White et al., 2018; Zhang et al., 2022; Zhang et al., 2021; White et al., 2016).

Mice homozygous for loss-of-function mutations in *Wnt5a* or *Ror2* recapitulate RS phenotypes, indicating causality (Yamaguchi et al., 1999; Alcantara et al., 2021; Gao et al., 2011). DVL family members have context-dependent roles in canonical, β-catenin-dependent Wnt signaling, and in the non-canonical Wnt PCP pathway, while ROR2 is considered as a non-canonical Wnt co-receptor that acts in concert with WNT5A to activate non-canonical signaling, and NXN activates the non-canonical pathway and inhibits β-catenin-mediated signaling (Funato and Miki, 2010; Gao et al., 2011; Rim et al., 2022).

Recessive OMOD (OMOD type 1/OMOD1) is associated with homozygous mutation of the glypican 6 (*GPC6*) gene and differs from RS and dominant OMOD (OMOD type 2/OMOD2) in that it reliably correlates with short stature among other differential features (Bayat et al., 2020; Arabzadeh et al., 2022). OMOD2 is associated with mutations in the *FZD2* gene. These include a mis-sense mutation in a conserved residue in the FZD domain upstream of all the DVL-interacting domains (p.Gly434Ser/Val) that is likely to alter their 3D structure (White et al., 2018); a mutation in the FZD-like N-terminus domain (p.Phe130Cysfs*98) that deletes all of the downstream sequences; a truncating mutation in the FZD domain upstream of all the DVL-interacting domains (p.Trp377*); and truncating mutations in the final DVL-interacting domain (p.Trp547* or p.Trp548*) (Nagasaki et al., 2018; Saal et al., 2015; Turkmen et al., 2017; Warren et al., 2018; White et al., 2018; Zhang et al., 2022). Although all of these mutations have dominant effects in humans, the mis-sense mutation p.Gly434Ser/Val correlates with the most-severe phenotypes (White et al., 2018; Zhang et al., 2022). A recent analysis of the spectrum of phenotypes associated with OMOD2 and *FZD2* mutations concluded that these are clinically indistinguishable from AD-RS and that OMOD2 should rather be considered as FZD2-associated AD-RS, contributing to ∼14% of all RS cases (Zhang et al., 2022; Zhang et al., 2021). Hereafter, we therefore refer to this syndrome as AD-RS/OMOD2.

The observations summarized above suggest involvement of FZD2 in regulating limb development and specifically implicate importance of the DVL-interacting domains. However, this hypothesis has not been tested through loss-of-function studies in a genetically manipulable model system. Furthermore, whether FZD2 activates canonical or non-canonical signaling during limb development is unclear.

To address these questions, we generated mice harboring a single-nucleotide insertion in the final DVL-interacting domain of *Fzd2* (*Fzd2^em1Smill^*, hereafter referred to as *Fzd2^INS^*). This mutation is predicted to truncate the FZD2 protein in the final DVL-interacting domain and to add an unrelated sequence of 39 amino acids. *Fzd2^INS^* mice mimicked the phenotypes seen in RS and OMOD2 patients, indicating that *FZD2* mutations are causative. We also generated mice carrying *Prx1-Cre* that is active in limb mesenchyme together with a conditional *Fzd2^fl^* allele that, in the presence of Cre recombinase, produces an antisense transcript, resulting in decreased levels of mRNAs for *Fzd2* and the related genes *Fzd1* and *Fzd7* that are also expressed in limb mesenchyme (Summerhurst et al., 2008). We found that Fzd deficiency in limb mesenchyme caused formation of shortened bone elements and was associated with disruption of Wnt/β-catenin and WNT5A/PCP signaling. Taken together, these findings demonstrate that FZD2 controls limb development by mediating different Wnt signaling pathways.

## RESULTS

### *Fzd2* is expressed in the ectoderm and mesenchyme of developing limb buds

**Fig. 1.**
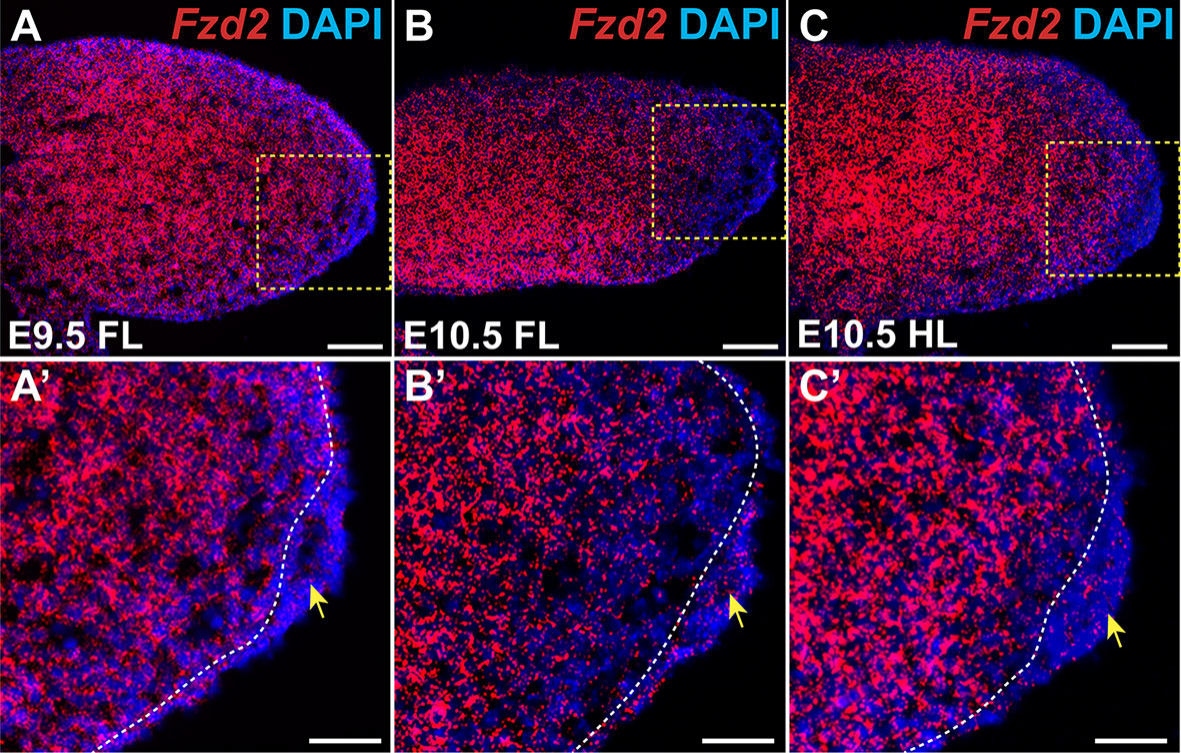
***Fzd2* is broadly expressed in the developing limb bud.** (A) RNAscope *in situ* hybridization (ISH) of sagittal sections shows that *Fzd2* is expressed in forelimb bud at E9.5. (A′) Magnified view of the indicated region in A. *Fzd2* expression is higher in the mesenchyme than in the ectoderm, including the apical ectodermal ridge (AER). (B) *Fzd2* expression persists in the ectoderm and mesenchyme of the forelimb bud at E10.5 but is decreased in distal mesenchyme compared with its expression at E9.5. (B′) Magnified view of the indicated region in B. (C) *Fzd2* expression in E10.5 hindlimb is similar to that in the forelimb. (C′) Magnified view of the indicated region in C. In all photomicrographs, dorsal limb is oriented at the top and distal to the right. Yellow arrows indicate the AER; white dashed lines mark the boundary between ectoderm and mesenchyme. *n*=3 samples analyzed per stage. Scale bars: 100 µm (A,B,C); 50 µm (A′,B′,C′).

To determine the location of *Fzd2* expression in developing mouse limb buds, we carried out *in situ* hybridization (ISH) for *Fzd2* mRNA at embryonic day (E)9.5 and E10.5. At E9.5, *Fzd2* was ubiquitously expressed in both ectoderm and mesenchyme of the emerging forelimb bud, with lower levels of expression in the ectoderm and in mesenchyme immediately underlying the AER, and most-intense expression in more proximal mesenchymal cells (Fig. 1A,A′). This expression pattern persisted in the forelimb bud at E10.5 (Fig. 1B,B′) and was similar in the E10.5 hindlimb bud (Fig. 1C,C′).

### An insertional mutation in the final DVL-interacting domain of FZD2 causes lethality of pups after birth

**Fig. 2.**
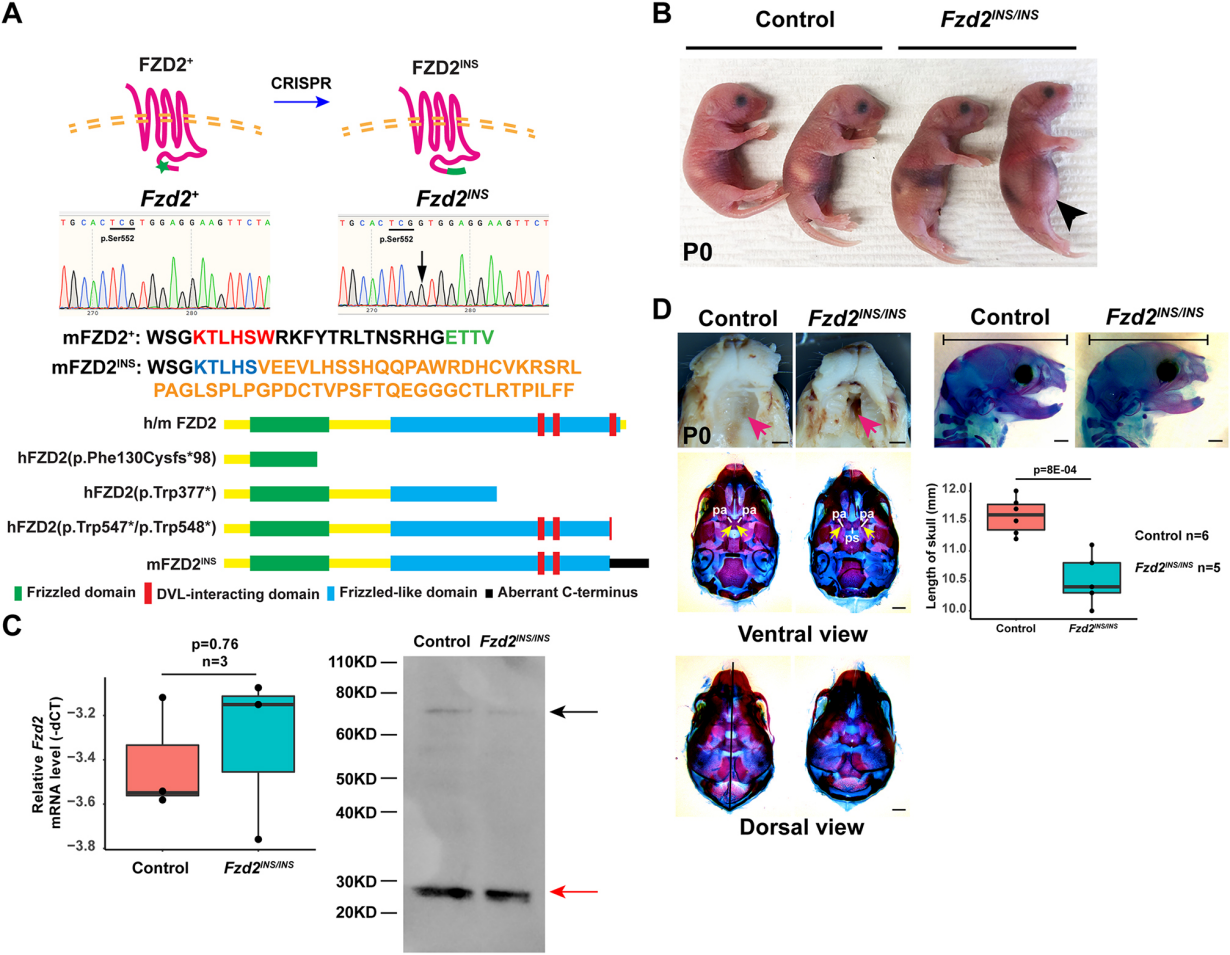
***Fzd2^INS/INS^* pups display craniofacial defects.** (A) CRISPR/Cas9 editing introduced an extra guanine (G) directly after p.Ser552, mutating the DVL-binding motif (red font), removing the PDZ-interacting domain (green font) and causing a frameshift in the C-terminus of FZD2, which produces an additional 39 amino acids (orange font). The schematic indicates the structure of wild-type human and mouse FZD2 (h/m FZD2) with the FZD domain indicated in green, the FZD-like domains indicated in blue, and the three DVL-interacting domains indicated in red. The predicted effects of human pathogenic FZD2 mutations hFZD2(p.Phe130Cysfs*98), hFZD2(p.Trp377*), hFZD2(p.Trp547*) and hFZD2(p.Trp548*), and the mouse *Fzd2^INS^* allele on protein structure are shown below. The aberrant C-terminus of the FZD2^INS^ protein is indicated in black. (B) *Fzd2^INS/INS^* neonates had swollen abdomens lacking milk spots (black arrowhead), compared with those of wild-type controls. (C) qPCR and immunoblotting showed that *Fzd2* mRNA and protein levels were similar in control and *Fzd2^INS/INS^* skin samples. The black arrow indicates FZD2 protein; the red arrow indicates a nonspecific band. (D) *Fzd2^INS/INS^* pups display severely clefted palates (pink arrows) and shortened skulls. Note the fusion of the bilateral palatal bones (pa) in control skull and separated palatal bones and visible presphenoid bone (ps) in *Fzd2^INS/INS^* skull (yellow arrows in ventral view). *Fzd2^INS/INS^* skulls did not display evidence of craniosynostosis. Quantitation of *Fzd2^INS/INS^* versus control skull lengths showed that *Fzd2^INS/INS^* skulls were statistically significantly shorter than control skulls (*n*=6 control and *n*=5 mutants analyzed). Littermate controls were wild type or *Fzd2^INS/+^*. Unpaired two-tailed Student's *t*-test was used to calculate *P*-values. *P*<0.05 was considered significant. For the box and whisker plots in C and D, the box represents the 25-75th percentiles, and the median is indicated. The whiskers show the minimum and maximum measurements. Scale bars: 1 mm.

To determine the functional consequences of truncating C-terminal FZD2 mutations observed in AD-RS/OMOD2 patients, we used CRISPR/Cas9 gene editing to generate a disruption in mouse *Fzd2*. The modified *Fzd2* allele (*Fzd2^INS^*) harbors an extra guanine between c.1656 and c.1657 (NM_020510.2: c.1656_1657insG), leading to a frameshift mutation that mutates the most C-terminal DVL-binding motif (KTxxxW) and removes the PDZ-interacting domain (ETTV), resulting in an aberrant C-terminus and a predicted protein that is 39 amino acids longer than wild-type FZD2 (Fig. 2A). The predicted effects of this mutation on mouse FZD2 protein compared with those of pathogenic mutations that truncate human FZD2 are diagrammed in Fig. 2A. *Fzd2^INS/+^* mice lacked limb abnormalities (Fig. S1A,B) or other overt phenotypes, and were fertile.

For comparison, we examined X-ray data publicly available on the International Mouse Phenotyping Consortium (IPMC) website for an independent predicted loss-of-function *Fzd2* mutant, *Fzd2^tm1.1(KOMP)Vlcg^*, that was generated via gene targeting (MGI:1888513) (Dickinson et al., 2016). Consistent with our data from *Fzd2^INS/+^* mice, measurements of ulna and tibia length in X-ray images showed no significant differences between 13-week-old *Fzd2^tm1.1(KOMP)Vlcg/+^* heterozygous mice and wild-type controls (*n*=6 male controls and *n*=6 male mutants) (Fig. S1C).

In contrast to the lack of phenotypes in mouse heterozygous *Fzd2^INS/+^* and *Fzd2^tm1.1(KOMP)Vlcg/+^* mutants, homozygous *Fzd2^INS/INS^* pups died within a few days of delivery and had extended abdomens containing air but little milk, suggesting difficulty in feeding (Fig. 2B). *Fzd2* mRNA and protein levels were comparable between control and *Fzd2^INS/INS^* skin samples, indicating that the *Fzd2^INS^* mutation did not result in nonsense-mediated mRNA decay or impaired translation (Fig. 2C). Detailed analysis revealed that the *Fzd2^INS/INS^* pups had a 100% penetrant phenotype of cleft palate and reduced distances from the back of the skull to the anterior tip of the upper jaw (*n*=5 mutants and *n*=6 controls analyzed; *P*=8×10^−4^) (Fig. 2D). Craniosynostosis was not observed in the mutants. These results were consistent with a prior study demonstrating a 50% penetrant cleft palate phenotype in mice carrying a hypomorphic mutation in *Fzd2* (Michalski et al., 2021 preprint; Yu et al., 2010). Data from our newly generated *Fzd2^INS/INS^* mice thus indicate that FZD2 deficiency alone is sufficient to cause abnormal craniofacial development.

### Limb development is defective in *Fzd2^INS/INS^* mice

**Fig. 3.**
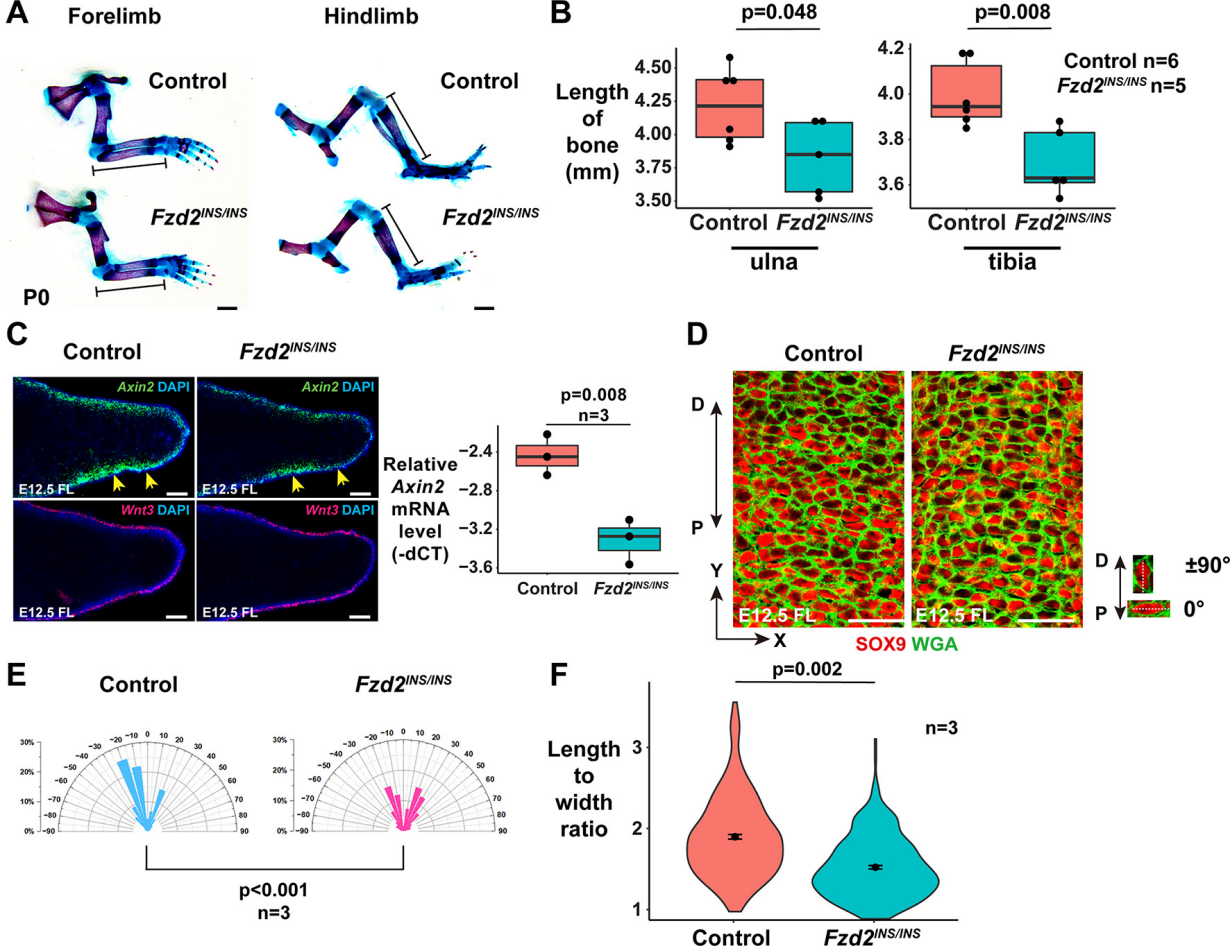
**The *FZD2^INS^* mutation causes defective limb development by altering both canonical Wnt and Wnt/PCP signaling.** (A) Skeletal preparations show that the ulna and tibia of *Fzd2^INS/INS^* pups are shorter than those of littermate controls at P0. (B) Quantitation of bone element lengths in *Fzd2^INS/INS^* pups (*n*=4 controls and *n*=3 mutants). Unpaired two-tailed Student's *t*-test was used to calculate *P*-values. *P*<0.05 was considered significant. (C) *Fzd2* deficiency causes reduced expression of *Axin2* (yellow arrows) but has little effect on *Wnt3* expression in forelimbs at E12.5 (left). qPCR shows that *Axin2* mRNA levels are statistically significantly reduced in E12.5 *Fzd2^INS/INS^* forelimb buds compared with those of controls (*n*=3 *Fzd2^INS/INS^* mutants and *n*=3 controls assayed) (right). A Kolmogorov–Smirnov test was used to calculate the *P*-value. (D) Wheat germ agglutinin (WGA; green) and SOX9 (red) staining show that elongation and orientation of digit chondrocytes is affected in *Fzd2^INS/INS^* mutants. (E) Quantitation of cell orientation in E12.5 forelimbs. One-hundred chondrocytes from each embryo were measured. Three pairs of control and mutant embryos were analyzed. The *x*-axis represents the angle of orientation; the *y*-axis represents the percentage of chondrocytes at angle X. Chondrocytes oriented horizontally are designated as 0°; chondrocytes oriented along the proximal–distal (P-D) axis are designated as ±90°. The Kolmogorov–Smirnov test was used to calculate the *P*-value. *P*<0.05 was considered significant. (F) Quantitation of the ratio of length to width of chondrocytes shows that this is significantly altered in E12.5 *Fzd2^INS/INS^* mutant forelimbs. One-hundred chondrocytes from each embryo and three embryos of each genotype were analyzed. Littermate controls were wild type or *Fzd2^INS/+^*. Unpaired two-tailed Student's *t*-test was used to calculate the *P*-value. *P*<0.05 was considered significant. For the box and whisker plots in B and C, the box represents the 25-75th percentiles, and the median is indicated. The whiskers show the minimum and maximum measurements. Scale bars: 1 mm (A); 100 µm (C); 25 µm (D).

*Fzd2^INS/INS^* pups did not show obvious limb patterning defects (Fig. 3A) but displayed statistically significantly shorter limb bones than those of littermate controls (Fig. 3B). To determine the mechanisms underlying limb defects in *Fzd2^INS/INS^* mice, we analyzed the effects of the *Fzd2^INS^* mutation on canonical and non-canonical Wnt signaling pathways. mRNA levels for *Wnt3*, which is expressed in limb ectoderm and directs canonical β-catenin-mediated signaling, were unaffected by the *Fzd2^INS^* mutation at E12.5; however, levels of canonical signaling, indicated by expression of the ubiquitous canonical Wnt target gene *Axin2* and assayed via ISH and quantitative PCR (qPCR), were downregulated in *Fzd2^INS/INS^* mutant limb buds compared with those of littermate controls (Fig. 3C), indicating that FZD2 mutation disrupts canonical signaling downstream of WNT3.

To assay for effects on non-canonical Wnt signaling, we measured the elongation and orientation of SOX9-expressing chondrocytes in distal digits, which is controlled by the WNT5A/PCP pathway (Yang et al., 2017). In E12.5 control digits, the majority of distal chondrocytes were elongated, and their major axes trended to be perpendicular to the P-D axis of the limbs; however, in *Fzd2^INS/INS^* digits, the elongation and orientation of chondrocytes was abnormal (Fig. 3D-F), similar to limb chondrocyte phenotypes in *Wnt5a^−/−^* embryos (Gao et al., 2011). Taken together, these data indicate that FZD2 mediates both canonical and WNT5A/PCP signaling pathways in limb development.

### Reduced Fzd gene expression in limb mesenchyme produces severe limb defects

As *Fzd2* is predominantly expressed in limb mesenchymal cells, we utilized *Fzd2^tm1Eem^* (hereafter designated as *Fzd2^fl^*) mice that permit conditional *Fzd2* recombination (Kadzik et al., 2014) to address whether mesenchymal *Fzd2* is required for normal limb development. We used *Prx1-Cre* (Logan et al., 2002) to drive recombination of the *Fzd2^fl^* allele in limb bud mesenchyme. We assessed the effects of mesenchymal *Fzd2^fl^* recombination in developing forelimb buds because *Prx1-Cre* drives mosaic deletion in hindlimb buds, which complicates analysis (Logan et al., 2002). Although originally described as a conventional floxed allele, a subsequent study (Michalski et al., 2021 preprint) and our data show that this *Fzd2^fl^* allele contains an inverted duplication of *Fzd2* with oppositely oriented *loxP* sites positioned respectively in the 3′ UTR and 5′ UTR of the duplicated genes. The 5′ UTR of the downstream copy of *Fzd2* contains a 92 bp deletion and an inserted 34 bp *loxP* sequence; thus, its predicted mRNA transcript is 58 bp shorter than that produced by the upstream *Fzd2* copy. To determine whether both copies of *Fzd2* are transcribed in the absence of Cre recombinase, we designed reverse transcription PCR (RT-PCR) primers in the 5′ UTR upstream of the 92 bp deleted region and in the coding sequence of *Fzd2* that are predicted to amplify a 526 bp fragment of the upstream *Fzd2* transcript and a 468 bp fragment of the downstream *Fzd2* transcript. The 526 bp fragment, but not the 468 bp fragment, was amplified in wild-type, *Fzd2^fl/+^* and *Fzd2^fl/fl^* mice, indicating that only the upstream *Fzd2* copy is transcribed (Fig. S2A). In line with this, *Fzd2^fl/fl^* mice did not display any overt pathology in the absence of Cre-mediated recombination. To confirm lack of limb phenotypes in *Fzd2^fl/fl^* mice, we assayed for these in detail and found that the lengths from the forelimb middle digit tip to the elbow, and the lengths of the hindlimb paws, were not statistically significantly different in *Fzd2^fl/fl^* compared with wild-type mice at postnatal day (P)7 (*n*=8 *Fzd2^fl/fl^* mutants and *n*=10 controls analyzed) (Fig. S2B). Taken together, these data indicate that the *Fzd2* allele is under the control of its normal *cis*-regulatory sequences in *Fzd2^fl^* mice, and that the coding sequence is intact and unmutated in the upstream copy of *Fzd2*.

Upon Cre-mediated recombination, the sequence between the two *loxP* sites in the *Fzd2^fl^* allele undergoes continuous inversion (Michalski et al., 2021 preprint) (Fig. S2C). This results in production of an inverted transcript complementary to *Fzd2* mRNA (Fig. S2C,D). The inverted transcript is likely to function as an antisense RNA and could potentially act *in trans* to bind to and degrade transcripts from the wild-type *Fzd2* allele in Cre-bearing *Fzd2^fl/+^* mutants via RNA interference (Katayama et al., 2005; Kumar and Carmichael, 1998). This observation also raised the possibility that the *Fzd2* antisense RNA could bind and degrade mRNAs for *Fzd1* and *Fzd7*, which are expressed in developing limb mesenchyme (Summerhurst et al., 2008) and show high sequence similarity with *Fzd2*.

**Fig. 4.**
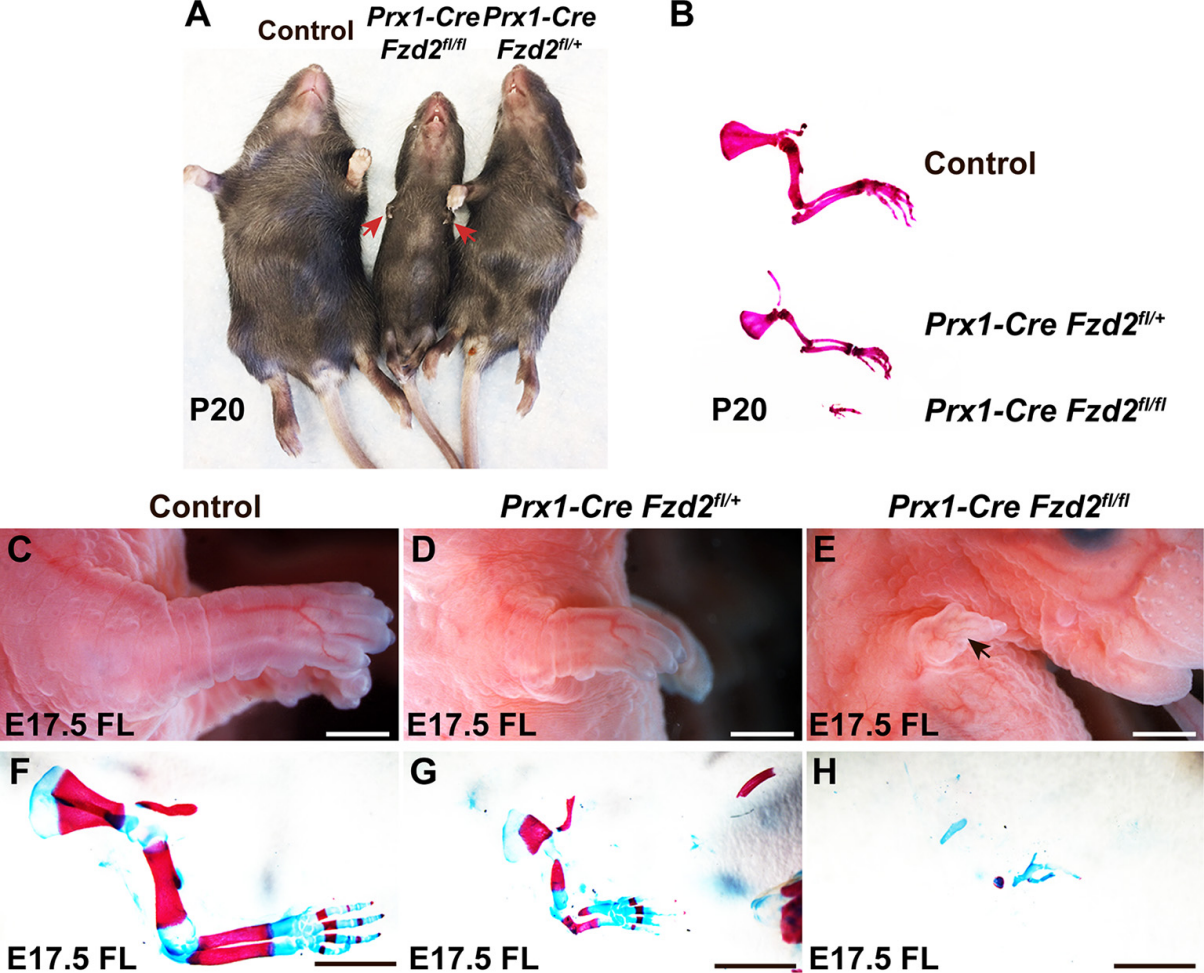
**Mesenchymal-specific recombination of *Fzd2^fl^* causes defective limb development.** (A) *Prx1-Cre Fzd2^fl/+^* and *Prx1-Cre Fzd2^fl/fl^* mutants have hypoplastic forelimbs at P20. (B) Skeletal preparation of P20 forelimbs. Compared with control forelimbs, *Prx1-Cre Fzd2^fl/+^* pups have hypoplastic forelimbs; *Prx1-Cre Fzd2^fl/fl^* pups only have a few residual bone elements in their forelimbs. (C-E) Whole-mount views of E17.5 control (C), *Prx1-Cre Fzd2^fl/+^* (D) and *Prx1-Cre Fzd2^fl/fl^* (E) forelimbs show reduced forelimb size in the *Prx1-Cre Fzd2^fl/+^* mutant (D) and a small residual forelimb in the *Prx1-Cre Fzd2^fl/fl^* mutant (E). (F-H) Skeletal preparations show that all the skeletal elements are hypomorphic in an E17.5 *Prx1-Cre Fzd2^fl/+^* mutant forelimb (G) and only a few skeletal elements develop in a *Prx1-Cre Fzd2^fl/fl^* mutant (H) compared with control (F). Controls were littermates lacking *Prx1-Cre* and/or *Fzd2^fl^*. Scale bars: 0.5 mm.

**Fig. 5.**
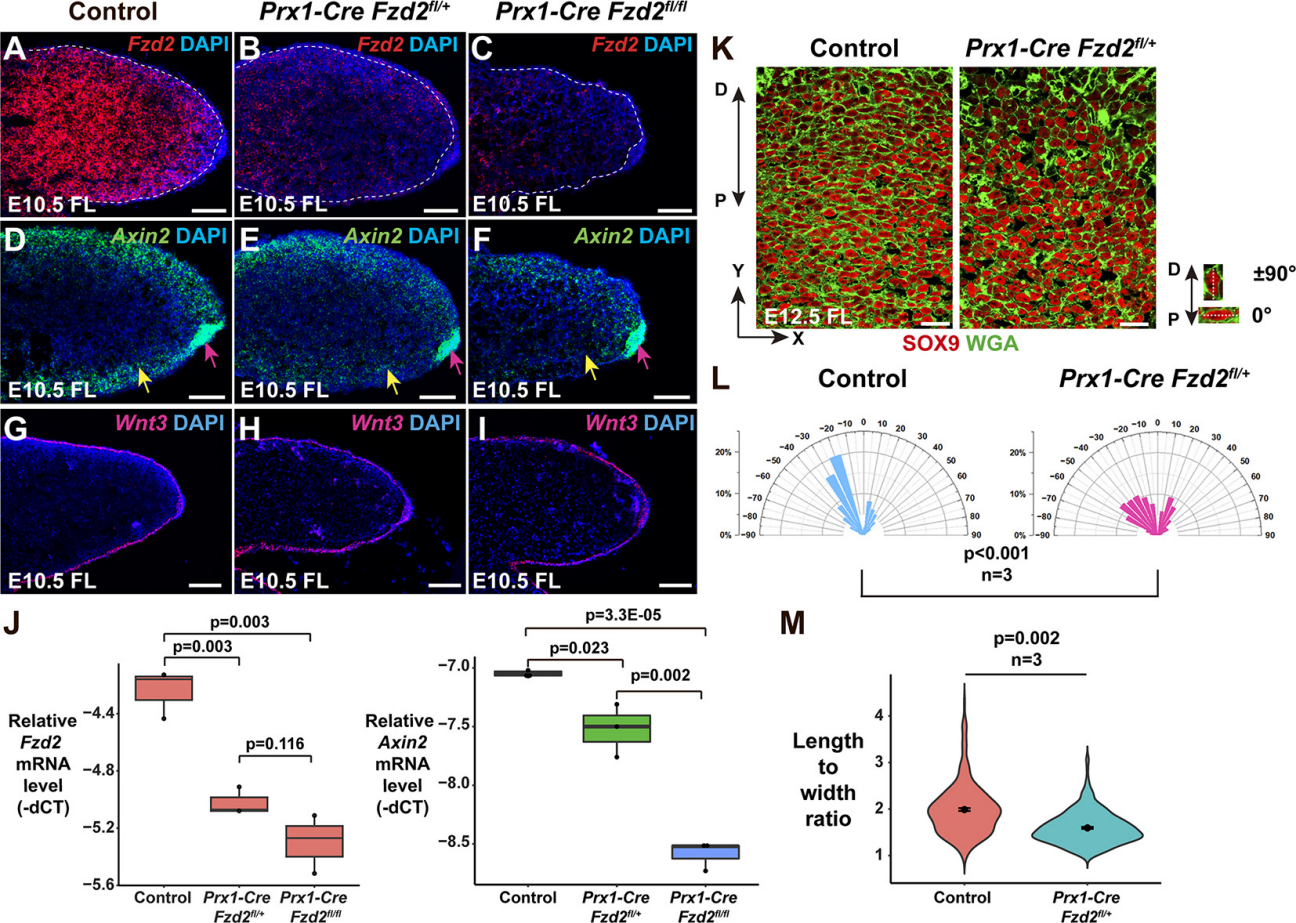
**Reduced mesenchymal Fzd expression affects both canonical and non-canonical Wnt signaling.** (A-C) ISH shows that mesenchymal *Fzd2* expression in E10.5 forelimb (FL) mesenchyme is reduced in *Prx1-Cre Fzd2^fl/+^* mutants (B) and almost absent in *Prx1-Cre Fzd2^fl/fl^* mutants (C) compared with control (A). (D-F) ISH shows that *Axin2* expression is reduced in distal forelimb mesenchyme (yellow arrows) but not in the AER (pink arrows) in E10.5 *Prx1-Cre Fzd2^fl/+^* (E) and *Prx1-Cre Fzd2^fl/fl^* (F) embryos compared with control embryos (D). (G-I) Ectodermal *Wnt3* expression is unaffected in the forelimbs of E10.5 *Prx1-Cre Fzd2^fl/+^* (H) and *Prx1-Cre Fzd2^fl/fl^* (I) embryos compared with control forelimbs (G). (J) qPCR quantification of *Fzd2* (left) and *Axin2* (right) mRNA levels in E10.5 forelimb mesenchyme shows statistically significantly decreased expression of *Fzd2* and *Axin2* expression in *Prx1-Cre Fzd2^fl/+^* and *Prx1-Cre Fzd2^fl/fl^* samples compared with samples from controls lacking *Prx1-Cre* or *Fzd2^fl^*. ‘−dCT’ refers to the delta between the cycle threshold (CT) for the target gene and the CT for *Gapdh*. *n*=3 per genotype. Unpaired two-tailed Student's *t*-test was used to calculate *P*-values. *P*<0.05 was considered significant. (K) WGA (green) stains cell membranes, revealing the shapes of SOX9 (red)-expressing chondrocytes. In E12.5 control forelimb digits, chondrocytes are elongated and predominantly lie perpendicular to the P-D axis; in *Prx1-Cre Fzd2^fl/+^* digits, the chondrocytes are more rounded and their orientation is more random. (L) Quantitation of chondrocyte orientation. At least 500 control and *Prx1-Cre Fzd2^fl/+^* chondrocytes from three embryos of each genotype were analyzed. The *x*-axis represents the angle of orientation; the *y*-axis represents the percentage of chondrocytes at each angle X. Chondrocytes oriented horizontally are designated as 0°; chondrocytes oriented along the P-D axis are designated as ±90°. A Kolmogorov–Smirnov test was used to calculate the *P*-value. *P*<0.05 was considered significant. (M) Quantitation of the length-to-width ratio of *Prx1-Cre Fzd2^fl/+^* chondrocytes compared with controls shows a statistically significant difference, demonstrating that chondrocyte cell shape is altered in the mutants. One-hundred chondrocytes from each embryo were analyzed from *n*=3 embryos per genotype. Unpaired two-tailed Student's *t*-test was used to calculate the *P*-value. *P*<0.05 was considered significant. For the box and whisker plots in J, the box represents the 25-75th percentiles, and the median is indicated. The whiskers show the minimum and maximum measurements. Controls were littermates lacking *Prx1-Cre* and/or *Fzd2^fl^*. Scale bars: 100 µm (A-I); 20 µm (K).

We found that *Prx1-Cre Fzd2^fl/+^* mutants were viable and fertile. Their forelimbs had normal digits but were shortened. More severe forelimb hypoplasia, with loss of almost all bone elements, was observed in postnatal *Prx1-Cre Fzd2^fl/fl^* mutants (Fig. 4A,B). Similar phenotypes were observed at E17.5 (Fig. 4C-H). ISH experiments confirmed that mesenchymal *Fzd2* mRNA levels were reduced in *Prx1-Cre Fzd2^fl/+^*, and almost absent in *Prx1-Cre Fzd2^fl/fl^*, forelimb mesenchyme during embryogenesis (Fig. 5A-C,J).

Levels of *Fzd2* mRNA were reduced more dramatically in *Prx1-Cre Fzd2^fl/+^* forelimb mesenchyme than would be expected in a conventional heterozygous mutant (Fig. 5A,B,J), consistent with degradation of transcripts from the wild-type allele. Additionally, we found that transcripts for *Fzd1* and *Fzd7* were statistically significantly downregulated in E11.5 *Prx1-Cre Fzd2^fl/+^* and *Prx1-Cre Fzd2^fl/fl^* forelimbs compared with the forelimbs of littermate controls (Fig. S2E). These data suggest that, although global homozygous loss of *Fzd2* alone is sufficient to produce limb phenotypes (Fig. 3A-F), limb phenotypes in *Prx1-Cre Fzd2^fl/+^* and *Prx1-Cre Fzd2^fl/fl^* mice could be a combined effect of reduced *Fzd2*, *Fzd1* and *Fzd7* expression in limb bud mesenchyme.

### Fzd genes mediate both canonical and non-canonical Wnt signaling in forelimb mesenchyme

As mesenchymal canonical Wnt/β-catenin signaling is required for limb development (Hill et al., 2006), we asked whether the Wnt/β-catenin pathway is affected in *Prx1-Cre Fzd2^fl/+^* and *Prx1-Cre Fzd2^fl/fl^* mutant mesenchyme. Expression of *Axin2*, a ubiquitous target of canonical signaling, was attenuated in a dose-dependent manner in limb bud mesenchyme, but not in the ectoderm, upon mesenchymal *Fzd2^fl^* recombination (Fig. 5D-F,J). Ectodermal *Wnt3* expression was not affected (Fig. 5G-I), indicating that mesenchymal FZD receptors mediate β-catenin signaling in the mesenchyme downstream of ectodermal WNT3.

We tested whether the non-canonical Wnt pathway is also affected upon mesenchymal *Fzd2^fl^* recombination by assaying elongation and orientation of SOX9-expressing chondrocytes in the distal digits, which is controlled by WNT5A/PCP signaling (Yang et al., 2017). As most bone elements are missing in *Prx1-Cre Fzd2^fl/fl^* forelimbs, we assayed for digit chondrocyte elongation and orientation in E12.5 *Prx1-Cre Fzd2^fl/+^* forelimbs. These experiments showed that digit chondrocyte elongation and orientation was abnormal in *Prx1-Cre Fzd2^fl/+^* mutants compared with controls (Fig. 5K-M), similar to the phenotypes observed in *Wnt5a^−/−^* embryos (Gao et al., 2011) and in *Fzd2^INS/INS^* mutants (Fig. 3D-F). Taken together, these observations indicate that FZD receptors mediate both Wnt/β-catenin and WNT5A/PCP pathways in embryonic forelimb mesenchyme.

## DISCUSSION

Dominant *FZD2* mutations are associated with AD-RS/OMOD2, but whether these are causative and the precise mechanisms by which FZD2 might act to control limb development have been unclear. Here, we show that limb and craniofacial defects observed in AD-RS/OMOD2 are phenocopied in mice carrying an insertional allele that, similarly to human pathogenic C-terminus truncating mutations p.Trp547* and p.Trp548* (Zhang et al., 2022), disrupts the final DVL-interacting domain of FZD2. These data provide definitive evidence for causality of *FZD2* mutations in human patients.

In addition to *FZD2* mutations, mutations in the non-canonical Wnt pathway components *WNT5A* and *ROR2* are associated with RS, suggesting that disruption of a non-canonical WNT5A–FZD2–ROR2 pathway might underlie defective limb development (White et al., 2018). In line with this, we find that chondrocyte elongation and orientation, which are controlled by non-canonical Wnt signaling, are disrupted in limb bud mesenchyme of *Fzd2^INS^* mice and in mice with mesenchymal-specific recombination of the *Fzd2^fl^* allele. Thus, disruption of the non-canonical pathway can account, at least in part, for limb phenotypes in RS.

Interestingly, however, *in vitro* experiments showed that FZD2 protein carrying the human FZD2 pathogenic mutation p.Trp548* was unable to transduce WNT3A-triggered canonical Wnt signaling (Saal et al., 2015), suggesting that impaired Wnt/β-catenin signaling might also contribute to defective limb development in these patients. In line with this, we observed decreased canonical Wnt signaling in the limb buds of *Fzd2^INS/INS^* embryos.

We found that *Prx1-Cre Fzd2^fl/+^* mice displayed limb shortening like that seen in RS/OMOD2 patients. The *Fzd2^fl^* allele produces an antisense transcript in the presence of Cre recombinase that likely targets transcripts from the wild-type *Fzd2* allele. Additionally, the *Fzd2* antisense transcript appears to cause degradation of transcripts from the closely related *Fzd1* and *Fzd7* genes in limb bud mesenchyme. These observations could explain why phenotypes were observed in *Prx1-Cre Fzd2^fl/+^* mice but not in mice heterozygous for *Fzd2^INS^* or *Fzd2^tm1.1(KOMP)Vlcg^*. Limb bone shortening was much more severe in *Prx1-Cre Fzd2^fl/fl^* mice than in *Prx1-Cre Fzd2^fl/+^* mice, and mice carrying either *Prx1-Cre Fzd2^fl/+^* or *Prx1-Cre Fzd2^fl/fl^* exhibited decreased canonical Wnt signaling activity in limb bud mesenchyme. These effects are also observed when β-catenin is deleted using the same Cre line (Hill et al., 2006). In addition, *Prx1-Cre Fzd2^fl/+^* mice showed striking defects in chondrocyte elongation and polarization, indicating that non-canonical Wnt signaling was disrupted in limb bud mesenchyme when mesenchymal FZD function was deficient. From these data, we can conclude that mesenchymal FZD receptors regulate limb development through both canonical and non-canonical Wnt signaling. However, elucidating the precise contribution of FZD2 in the mesenchyme must await analysis of a conventional floxed allele.

Taken together, data from the *Fzd2^INS/INS^* and *Prx1-Cre Fzd2^fl^* mutants suggest that shortened limbs observed in FZD2-associated AD-RS/OMOD2 patients result from FZD2 deficiency in developing limb bud and are caused by defects in both canonical and non-canonical Wnt signaling.

We noted that, in contrast to the dominant nature of human pathogenic *FZD2* mutations, mice heterozygous for *Fzd2^INS^* did not display obvious limb defects or aberrant canonical and non-canonical Wnt signaling; these were only apparent in homozygous *Fzd2^INS/INS^* mutants. Similarly, our analysis of publicly available data for mice heterozygous for an independent predicted loss-of-function *Fzd2* mutation, *Fzd2^tm1.1(KOMP)Vlcg^* (Dickinson et al., 2016), revealed no significant limb abnormalities compared with wild-type littermates. Possible explanations for these apparent differences in gene dosage requirement between mice and human patients include that (1) the mouse *Fzd2^tm1.1(KOMP)Vlcg^* and *Fzd2^INS^* mutations are both hypomorphs; (2) the human mutations have dominant-negative effects, perhaps by resulting in production of truncated FZD proteins that bind Wnt ligands but cannot activate downstream signaling; or (3) *FZD2* is haplo-insufficient in humans, but not in mice, possibly due to modifying mutations at other loci in humans, or to developmental compensation in mice. Regarding the latter, it is interesting to note that WNT5A loss-of-function mutations have dominant effects in human RS patients but are recessive in mice (Yamaguchi et al., 1999; Person et al., 2010). Further analyses will be required to distinguish among these scenarios. In summary, although at least seven Fzd genes are expressed in the developing mouse limb bud (Summerhurst et al., 2008), the functions of individual FZD family members in this context have been unclear. Our data identify FZD2 as a key component of both canonical and non-canonical Wnt signaling pathways in limb development and provide a mechanistic understanding of the defects in this process that are observed in patients carrying *FZD2* mutations. These findings will inform future research aimed at developing therapeutic interventions for AD-RS/OMOD2 patients.

## MATERIALS AND METHODS

### Mice

The following mouse lines were used: *Fzd2^fl^* (Kadzik et al., 2014), *Prx1-Cre* (The Jackson Laboratory, strain #005584) and *K14-Cre* (The Jackson Laboratory, strain #018964). All mice were maintained on a mixed strain background. Mice were allocated to experimental or control groups according to their genotypes, with control mice being included in each experiment. Male mice carrying *Prx1-Cre* were crossed with *Fzd2^fl/fl^* females to avoid potential germ line recombination. Mice were included in the analysis based on their genotypes. Mice were not randomized, as genotype information was required to assign them to control and experimental groups. Investigators were aware of genotype during allocation and animal handling as this information was required for appropriate allocation and handling. Immunostaining and ISH studies were carried out, and data were recorded in a manner that avoided observer bias. Up to five mice were maintained per cage in a specific pathogen-free barrier facility on standard rodent laboratory chow (Purina, 5001). All animal experiments were performed under approved animal protocols according to institutional guidelines established by the Icahn School of Medicine at Mount Sinai IACUC committee.

### Generation of CRISPR mutant mice

Female C57BL/6 mice (6-8 weeks old) were superovulated by intraperitoneal injection of 5 IU of pregnant mare serum gonadotropin followed 48 h later by 5 IU of human chorionic gonadotropin and were mated to B6D2F1 males. A cocktail solution containing 50 ng/µl gRNA (Integrated DNA Technologies; 5′-ACACTCGTCTCACCAACAGCCGG-3′) and 100 ng/µl Cas9 mRNA (Thermo Fisher Scientific, A29378) was injected into one blastomere of two-cell embryos so that resulting embryos would be mosaic for the *Fzd2* mutation with at least 50% of the cells being wild type. This approach was taken because injection into one-cell embryos was found to yield only homozygous mutants that were perinatal lethal and so could not be used to establish mutant lines. Embryos were incubated at 37°C, 5% CO_2_ in KSOM medium (Millipore, MR-202P-5F). The KSOM culture drops were covered with mineral oil (Millipore, ES-005-C) to prevent evaporation. After cocktail injection, the two-cell embryos were transferred the same day into the oviducts of Swiss Webster E0.5 pseudopregnant recipient females, which were synchronized by using Swiss Webster vasectomized males. Micromanipulation, embryo collection and embryo transfers were performed at room temperature in HEPES-buffered CZB medium. Founder mice were crossed with wild-type C57BL/6 mice to yield *Fzd2^INS/+^* offspring. *Fzd2^INS/+^* male and female mice were intercrossed to produce *Fzd2^INS/+^*, *Fzd2^INS/INS^* and *Fzd2^+/+^* mice for analysis. Genotyping was performed by PCR using primers *Fzd2^INS^*-F, 5′-CACGACGGCACCAAGACGGA-3′; *Fzd2^INS^*-R, 5′-GAGACCGCTTCACACAGTG-3′. The PCR product was then sequenced using primer *Fzd2^INS^*-F.

### RNA ISH

Embryos at the indicated stages were harvested and fixed with 4% paraformaldehyde (PFA; Affymetrix/USB) in PBS overnight at 4°C. RNAscope was performed on fixed frozen sections following the user's guide provided by Advanced Cell Diagnostic (ACD) using probes for *Fzd2* (ACD, 565781), *Wnt3* (ACD, 312241) and *Axin2* (ACD, 400331). The sections were observed and photographed using a Leica Microsystems DM5500B fluorescent microscope.

### Skeletal preparation with Alcian Blue and Alizarin Red staining

Euthanized embryos or pups were skinned, eviscerated and fixed with 100% ethanol for 48 h and transferred to acetone for 24 h. The samples were placed in staining solution containing 0.015% Alcian Blue and 0.005% Alizarin Red for 1 week, and then treated with 1% KOH/10% glycerol until clear. Skeletal samples were photographed in 70% glycerol solution.

### Quantification of chondrocyte orientation and shape

E12.5 forelimbs were fixed in 4% PFA overnight at 4°C, embedded in OCT and sectioned at 10 µm. Sections were incubated with anti-SOX9 antibody (Millipore, AB5535; 1:200) (Lefebvre et al., 1997) overnight at 4°C, followed by incubation with Alexa Fluor-labeled secondary antibodies (Thermo Fisher Scientific), and were washed with phosphate buffered saline with 0.1% Tween (PBST). The sections were co-stained with wheat germ agglutinin (WGA) according to the manufacturer's instructions (Biotium). Sections were photographed using a TCS SP8 confocal microscope (Leica Microsystems). The orientation of SOX9-expressing chondrocytes in the middle digit was determined by measuring the angle between the *x*-axis and the major axis of the chondrocyte. Analysis of chondrocyte shape was performed manually by measuring the lengths of the major and minor axes of individual cells using ImageJ and following the software user instructions. Data were analyzed by ImageJ (v1.49, National Institutes of Health) and plotted using Microsoft Excel and R studio with ggplot2.

### qPCR

Limb buds at E10.5 or E11.5 were dissected and incubated in Dispase II (Gibco) solution for 30 min at 37°C, and ectoderm was separated from the mesenchyme with forceps. Total RNA was extracted from E10.5 and E11.5 limb bud mesenchyme, or from whole limb buds at E12.5, using TRIzol (Thermo Fisher Scientific), purified using an RNeasy kit (Qiagen) and treated with an RNase-free DNase kit (Qiagen). Reverse transcription was performed using a High-Capacity cDNA Reverse Transcription Kit (Applied Biosystems), and cDNA was subjected to real-time PCR using the StepOnePlus system and SYBR Green Kit (Applied Biosystems). *Gapdh* was used as an internal control, and expression differences were determined using the −ΔCT method. Primers for *Gapdh* were *Gapdh*-F: 5′-GAGAGGCCCTATCCCAACTC-3′; *Gapdh*-R: 5′-GTGGGTGCAGCGAACTTTAT-3′. Primers for *Axin2* were *Axin2*-F, 5′- GCTGGTTGTCACCTACTTTTTCTGT-3′; *Axin2*-R, 5′-GGGGAGCACTGTCTCGTCGTC-3′. Primers for *Fzd2* were *Fzd2*-F, 5′-CTTCACGGTCACCACCTATTT-3′; *Fzd2*-R, 5′-AACGAAGCCCGCAATGTA-3′. Primers for *Fzd1* were *Fzd1*-F, 5′-TGCTTTGGTTGCTGGAGGCT-3′; *Fzd1*-R, 5′-CCGTTCGCCGTTGTACTGCT-3′. Primers for *Fzd7* were *Fzd7*-F, 5′-CCCATCCCACCCCCCTTG-3′; *Fzd7*-R, 5′-GATTTCTGTGGCTTTGCCTGTAA-3′.

### RT-PCR

Total RNA was extracted from keratinocytes of control and *K14-Cre Fzd2^fl/fl^* embryos at E14.5 using TRIzol (Thermo Fisher Scientific) and purified using an RNeasy kit (Qiagen). Total RNA samples were further treated with an RNase-free DNase kit (Qiagen), reverse transcribed using a High-Capacity cDNA Reverse Transcription Kit (Applied Biosystems), and cDNA was subjected to PCR. The following primers were used: *loxP*-F, 5′-GCCTGCTCGCTATTTTTGTTGGC-3′; *loxP*-R, 5′-AAATGAGGAGGGAGAAAGAGGGGG-3′; *Fzd2*-R: 5′-AACGAAGCCCGCAATGTA-3′; 5′ UTR-F, 5′-GTGAGGGCTGAAGGAGGCAC-3′; CDS-R, 5′-GCCAAGAAGGTTGGGCATGA-3′.

### Western blotting

The back skin of neonatal pups was collected and lysed with RIPA buffer (Santa Cruz Biotechnology). Western blotting was performed using an XCell SureLock Mini-Cell Electrophoresis System (Invitrogen). FZD2 was detected by anti-FZD2 antibody (Abcam, ab109094; 1:500) (Fu et al., 2020). Signal was detected and documented using a ChemiDoc MP system (Bio-Rad).

### Statistical analyses

Where possible, at least five samples were used for each experimental or control group. A sample size of *n*=5 provides 80% power at a two-sided significance level of 0.05 to detect a difference (effect size) of 2.0 s where *s* is the standard deviation. Statistical tests were chosen to be appropriate for the type of data. Unpaired two-tailed Student's *t*-test was used to calculate statistical significance for quantitation of bone element lengths and skull lengths, quantitation of ratios of chondrocyte length to width, and qPCR assay results. The Kolmogorov–Smirnov test was used to calculate statistical significance for quantitation of chondrocyte orientation. *P*<0.05 was considered significant.

## Supplementary Material

10.1242/dmm.049876_sup1 Supplementary information
