# Supplementary material for: FZD2 regulates limb development by mediating β-catenin-dependent and -independent Wnt signaling pathways
Source: Dis Model Mech. 2023 Mar 24;16(3):dmm049876. doi: 10.1242/dmm.049876 (PMC10073008; doi:10.1242/dmm.049876)
Supplement: Supplementary information [file dmm-16-049876-s1.pdf]

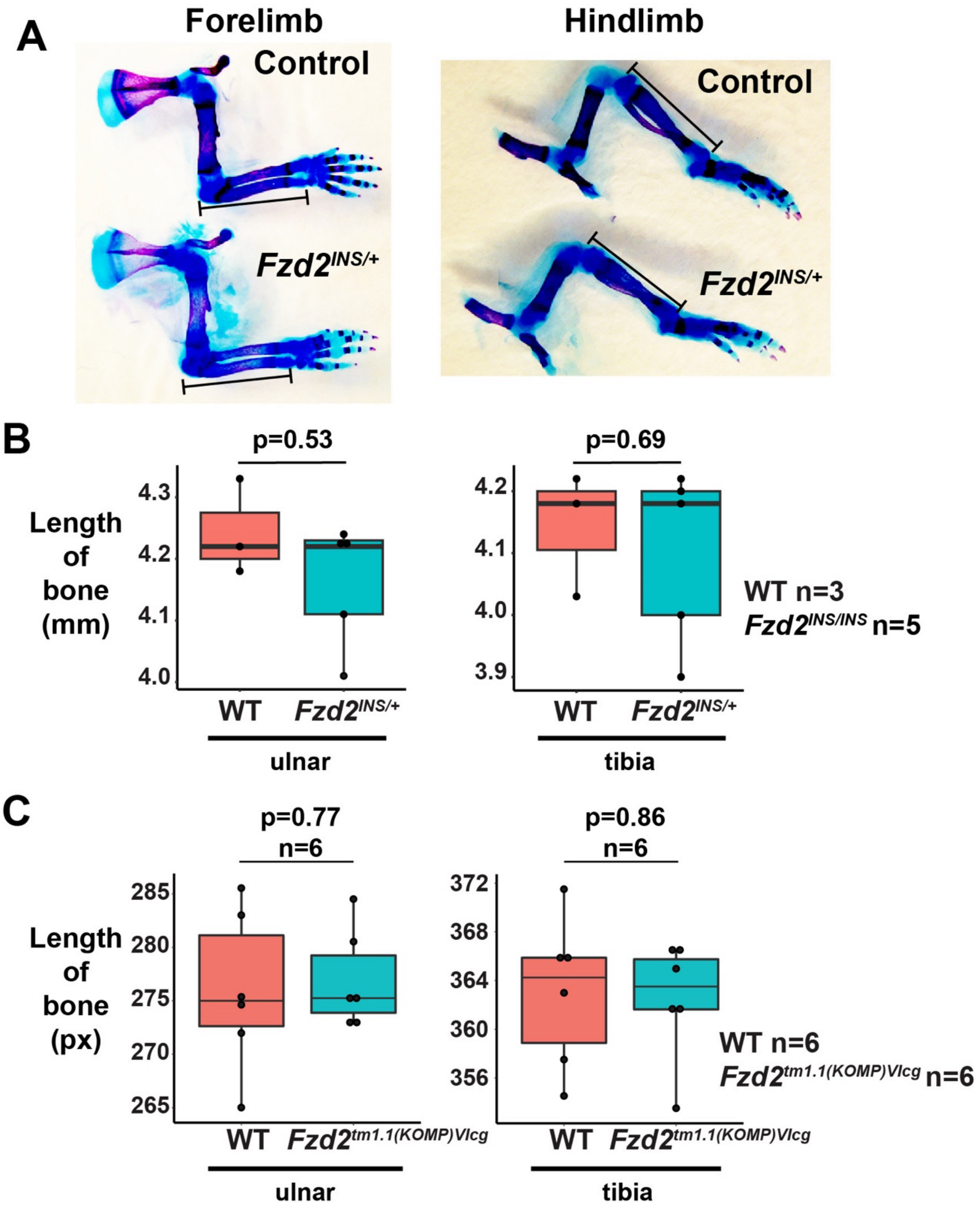

Fig. S1. The length of the ulna and tibia is not affected in heterozygous *Fzd2*$^{fl/+}$ or *Fzd2*$^{tm1.1(KOMP)Vlcg/+}$ mice. (A) Skeletal preparations show that the ulna and tibia of *Fzd2*$^{INS/+}$ pups are similar to those of wild type (WT) littermates at P0. (B) Quantitation of bone element lengths in *Fzd2*$^{INS/+}$ pups and wild type littermates (n=3 controls and 5 mutants). P-values were calculated with two-tailed Student's *t*-test. $p<0.05$ was considered significant. (C) X-ray images of *Fzd2*$^{tm1.1(KOMP)Vlcg/+}$ mice at 13-weeks of age were accessed at the IMPC website (https://www.mousephenotype.org/). The lengths of the ulna and tibia, measured using Image J (V1.49, NIH) and plotted using R studio with ggplot2, showed no significant difference between wild type and heterozygous *Fzd2*$^{tm1.1(KOMP)Vlcg}$ mice. N=6 male WT mice and n=6 male mutants were analyzed.

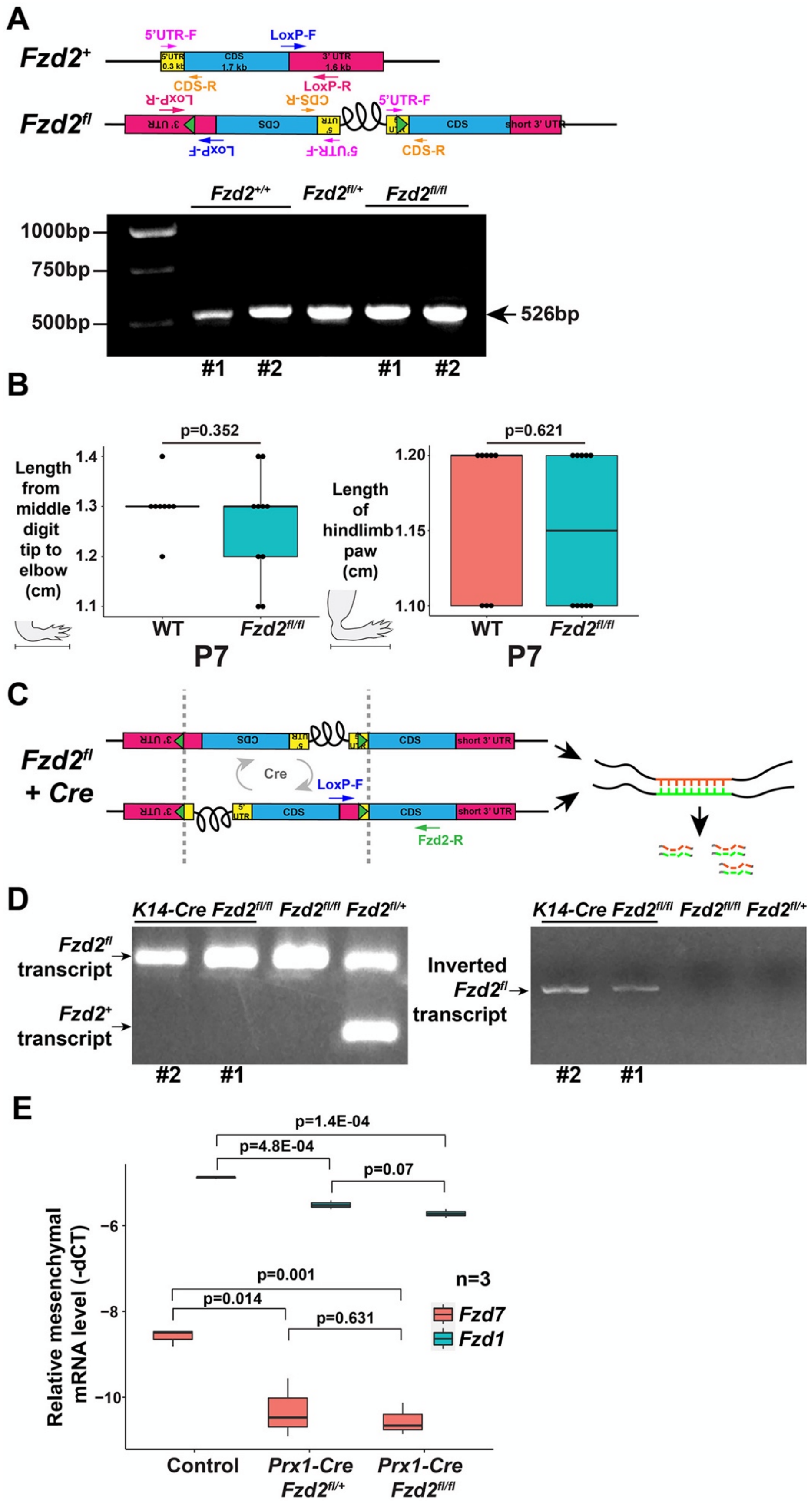

Disease Models & Mechanisms • Supplementary information

Fig. S2. The *Fzd2*^{fl} allele is a complex genomic alteration and generates antisense transcripts in the presence of Cre recombinase. (A) Schematic view of the wild type *Fzd2* allele (*Fzd2*$^{+}$) and the *Fzd2*^{fl} allele. In keratinocytes isolated from *Fzd2*$^{fl/+}$ and *Fzd2*$^{fl/fl}$ mice, the RT-PCR primer set 5'UTR-F and CDS-R amplifies the upstream copy of *Fzd2* resulting in a 526bp product; this product is also detected in wild-type keratinocytes. Transcripts from the downstream *Fzd2* copy in *Fzd2*$^{fl/+}$ and *Fzd2*$^{fl/fl}$ samples are predicted to yield a 468bp RT-PCR product using 5'UTR-F and CDS-R primers; however, as in wild-type samples, a 468bp product was not detected in *Fzd2*$^{fl/+}$ or *Fzd2*$^{fl/fl}$ keratinocytes, indicating that the downstream *Fzd2* copy is not transcribed. (B) Quantification of the length from the forelimb middle digit to the elbow, and the length of the hindlimb paw, shows that these are not significantly different between P7 *Fzd2*$^{fl/fl}$ and wild-type mice (n=8 wild-type and n=10 *Fzd2*$^{fl/fl}$ mice analyzed). (C) In the presence of Cre recombinase, the *Fzd2*^{fl} allele is predicted to undergo continuous inversion, producing complementary mRNAs that could potentially bind to each other to prevent normal *Fzd2* translation. (D) In *Fzd2*$^{fl/+}$ keratinocytes, both *Fzd2*$^{+}$ and *Fzd2*^{fl} mRNAs are detected by RT-PCR with primers *LoxP-F* and *LoxP-R* (see indicated positions of these primers in (A)). In *Fzd2*$^{fl/fl}$ keratinocytes, only *Fzd2*^{fl} transcripts are detected (left panel). In the presence of Cre recombinase, both antisense *Fzd2*^{fl} transcripts, amplified with primers *LoxP-F* and *LoxP-R* (left panel), and sense *Fzd2*^{fl} transcripts, amplified with primers *LoxP-F* and *Fzd2-R* (right panel; see indicated positions of these primers in (C)), are produced. (E) Expression of *Fzd1* (blue boxes) and *Fzd7* (red boxes), which are closely related to *Fzd2*, is reduced in E11.5 forelimb mesenchyme of *Prx1-Cre Fzd2*$^{fl/+}$ and *Prx1-Cre Fzd2*$^{fl/fl}$ mutants compared with control mice lacking *Prx1-Cre* or *Fzd2*^{fl}.

Disease Models & Mechanisms • Supplementary information
